# Immediate impact of COVID-19 on mental health and its associated factors among healthcare workers: A global perspective across 31 countries

**DOI:** 10.7189/jogh.10.020381

**Published:** 2020-12

**Authors:** Mila Nu Nu Htay, Roy Rillera Marzo, Ayesha AlRifai, Fatjona Kamberi, Radwa Abdullah El-Abasiri, Jeldah Mokeira Nyamache, Htet Aung Hlaing, Mayada Hassanein, Soe Moe, Tin Tin Su, Adinegara Lutfi Abas

**Affiliations:** 1 Department of Community Medicine, Melaka-Manipal Medical College, Manipal Academy of Higher Education (MAHE), Melaka, Malaysia; 2 Department of Public Health, Faculty of Medicine, Asia Metropolitan University, Johor, Malaysia; 3 Birzeit University & Arab American University, Palestine; 4 Research Center of Public Health, Faculty of Health, University of Vlore “Ismail Qemali”, Vlore, Albania; 5 Department of Pharmacy Practice and Clinical Pharmacy, Faculty of Pharmaceutical Sciences and Pharmaceutical Industries, Future University in Egypt, Cairo, Egypt; 6 North Star Alliance, Kenya, Uganda, Tanzania, Zimbabwe, Mozambique, and South Africa; 7 Pun Hlaing Siloam Hospital, Yangon, Myanmar; 8 Maternal and Child Health Unit, Alexandria Fever Hospital, Alexandria, Egypt; 9 South East Asia Community Observatory (SEACO) & Global Public Health, Jeffery Cheah School of Medicine and Health Sciences, Monash University Malaysia, Bandar Sunway, Malaysia

The emerging infectious disease, novel coronavirus (COVID-19) outbreak began in December 2019 and spread worldwide [1]. A total of 213 countries, areas, or territories were affected in April 2020 [2]. Coronaviruses are known to cause respiratory tract infection among humans, similar to the previous outbreak of Middle East respiratory syndrome coronavirus (MERS-CoV) [3] and Severe Acute Respiratory Syndrome (SARS) [4]. Severe acute respiratory syndrome coronavirus 2 (SARS-CoV-2) was unknown to be an infectious agent for humans before the outbreak in December 2019. The patients may mainly be present with fever, dry cough, and respiratory problems, while 80% of the infected cases are mild or asymptomatic [5].

The increasing numbers of infectious cases overwhelmed the workload in healthcare sectors in different countries. Although the social distancing and stay at home advice are recommended in the community, the healthcare workers were continuing to work in the respective areas [6]. During the pandemic, healthcare workers were at high risk of infection, having physical exhaustion, and an impact on their mental health due to the loss of the infected patients, personal safety, concern of passing infections to family members [6].

Although a few studies have been conducted in China, little is known about the worldwide situation and comparison on mental health impact among healthcare workers due to the COVID-19 pandemic and its associated factors. This study investigated the immediate impact of COVID-19 pandemic on the mental health of healthcare workers in terms of anxiety, depression, and associated factors with mental health issues.

We investigated the immediate impact on mental health by recruiting the respondents from various healthcare professions, including doctors, nurses, midwives, medical assistants, laboratory technicians, medical educators, public health practitioners, who were working at either government or private sectors between 20 April 2020 and 21 May 2020. The country representative researchers from Albania, Egypt, Iraq, Kenya, Mozambique, Myanmar, Palestine, Philippines, South Africa, Tanzania, Uganda, and Zimbabwe spread the web-based questionnaire. Our study investigated the mental health impact of COVID-19 especially in the low- and middle-income countries, where the healthcare systems had limited resources to tackle the current pandemic, and mental health support could be sparse. On the first day of data collection by using web-based survey, the COVID-19 situation in those countries was at the beginning stage of spread [7]. Although there are representative countries, respondents were not limited by nationalities but were allowed to participate from all countries around the world. The respondents were recruited with non-random convenience sampling method. Participation was voluntary and informed consent was obtained from the respondents. Ethical approval was granted from the Research Ethics Committee from Asia Metropolitan University (AMU), Malaysia, Project Ref No: AMU/MREC/FOM/NF/03/2020.

The demographic information on nationality, sex, age, religion, marital status, living status during COVID-19 pandemic, and work-related questions were included in the survey. The outcome measures were assessed by using the Generalized Anxiety Disorder (GAD-7) scale [8], and the Patient Health Questionnaire (PHQ-9) [9]. The GAD-7 is a brief instrument to screen the generalized anxiety disorder, which demonstrated good psychometric properties [8]. The validity and reliability of the GAD-7 had been proven across the various population [10-12]. To measure the severity of depression, the PHQ-9 was used in this study, which is relevant to apply in both clinical and research contexts [9,13]. The PHQ-9 had been proven its validity and reliability across the population [14,15] (Ref S1 in the **Online Supplementary Document**). Both GAD-7 and PHQ-9 were valid, reliable and short scales to assess the mental health issues of anxiety and depression, and therefore, these measures were selected as study instruments for data collection.

The original English version of the questionnaire was used for data collection. The decision was made based on consultation with the contact person of each country concerning English language competency of healthcare workers in the respective country. However, exception was made for Albania where the questionnaire was translated to the Albanian language by forward- and backward-translation following the method of linguistic validation (Ref S2 in the **Online Supplementary Document**).

The data from surveys were pooled into a combined data set and analysed by using SPSS (IBM SPSS Statistics for Windows, Version 23.0. IBM Corp, Armonk, NY, USA). The descriptive analysis was carried out, the frequency and percentage of demographic and occupational-related variables were presented in the tables. The prevalence of the outcomes was analysed. To identify the factors associated with anxiety among health workers, bivariate and multivariate analyses were performed. The χ^2^ (chi-square test) was used to examine the nature of the association between the anxiety and depression with occupation, sex, health provider treating COVID-19 cases and availability of mental health support team at workplace. The logistic regression analysis was applied to investigate the factors that explained and predicted the anxiety and depression. Instead of the linear probability model, the logistic regression function is preferable to fit some kinds of sigmoid curves when the response variable is dichotomous, and that reasonably portrays the reality of outcome events (Ref S3 in the **Online Supplementary Document**. The cutoff score of having generalized anxiety disorder [8] and depression symptoms [10] is 10, respectively. The response score was coded dichotomically, ie, those who had used anxiety and depression were coded as “1” and those who had not as “0.” The odds ratios and their 95 percent CI were calculated. *P* < 0.05 was considered to be statistically significant.

**Figure:**
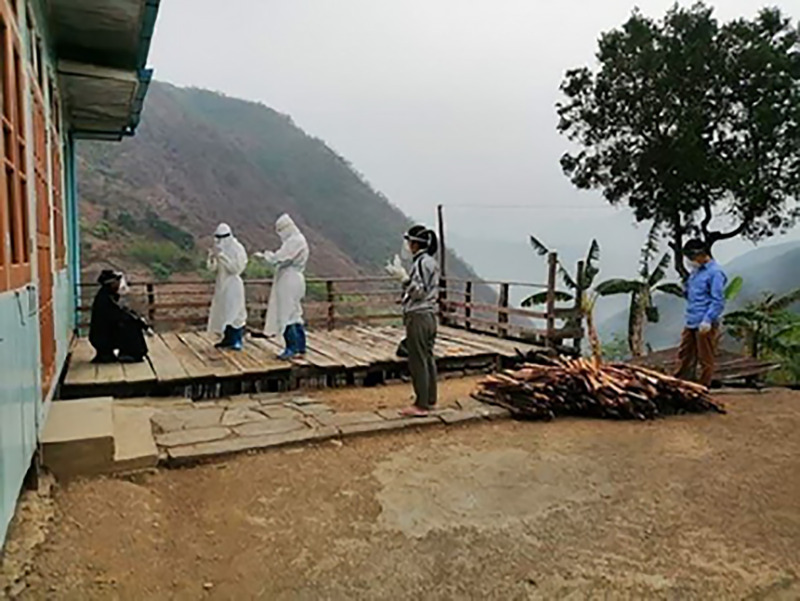
Photo: Frontline healthcare workers working at a village in Myanmar during COVID-19 pandemic (used with permission).

## IMMEDIATE IMPACT OF COVID-19 ON MENTAL HEALTH AMONG HEALTHCARE WORKERS

A total of 2166 respondents from 31 countries worldwide responded to the survey. Among them, 69 respondents’ data were incomplete and therefore, were excluded from the analysis. A total of 2097 respondents were included in the analysis. The participant’s geographic distributions were described in **Table 1**. Approximately half of them were living in the Eastern Mediterranean Region (EMRO) (52.0%), and a quarter was from the Western Pacific Region (WPRO) (25.4%) (Ref S4 in the **Online Supplementary Document**). The majority of the respondents, 79.2%, were from the lower-middle-income countries according to the world bank classification (Ref S5 in the **Online Supplementary Document**) (**Table 1**).

**Table 1:** Respondents’ geographic location according to the World Health Organization Regions and World Bank country classification on income (n = 2097)

	List of countries and territories of respondents	No. (%)
**World Health Organization Regions (Ref S4 in the Online Supplementary Document)**		
Eastern Mediterranean Region (EMRO)	Cyprus, Egypt, Iraq, Lebanon, Pakistan, Palestine, Syria	1091 (52.0)
Western Pacific Region (WPRO)	Japan, Philippines, Republic of Korea	532 (25.4)
European Region (EURO)	Albania, Germany, Italy, Sweden, United Kingdom	337 (16.1)
South-East Asian Region (SEARO)	Bangladesh, India, Myanmar, Nepal, Thailand	88 (4.2)
African Region (AFRO) and Region of the Americas (PAHO)	Ethiopia, Kenya, Lesotho, Mozambique, Nigeria, Rwanda, South Africa, Suriname, Tanzania, Uganda, Zimbabwe	49 (2.3)
**World Bank country classification by income (Ref S5 in the Online Supplementary Document)**		
Low-income countries	Ethiopia, Mozambique, Nepal, Rwanda, Syria, Tanzania, Uganda	30 (1.4)
Lower-middle-income countries	Bangladesh, Egypt, India, Kenya, Lesotho, Myanmar, Nigeria, Pakistan, Palestine, Philippines, Zimbabwe,	1661 (79.2)
Upper-middle-income countries	Albania, Iraq, Lebanon, South Africa, Suriname, Thailand	391 (18.7)
High-income countries	Cyprus, Germany, Italy, Japan, Republic of Korea, Sweden, United Kingdom	15 (0.7)

Table S1 in the **Online Supplementary Document** represents the socio-demographic characteristics and occupations of respondents. Approximately one third of respondents were doctors (36.72%), meanwhile, 22.84% were nurses and 40.44% were other healthcare workers (Table S1 in the **Online Supplementary Document**).

Table S2 in the **Online Supplementary Document** presents the prevalence of anxiety and depression among healthcare workers. Overall, the prevalence of anxiety among study respondents was 60%.

Table S2 in the **Online Supplementary Document** also presents the prevalence of depression. Overall, the prevalence of depression among study respondents was 53%.

## FACTORS ASSOCIATED WITH ANXIETY AND DEPRESSION AMONG HEALTHCARE WORKERS

Table S3 in the **Online Supplementary Document** describes the relationship between risk factors for anxiety and adjustment for age, sex, religion, marital status, occupation, work experience, staying ‘alone or with family/friends or colleagues,’ and working in the intensive care unit (ICU) and current workplace. The healthcare workers staying with family and friends were at lower risk of anxiety compared to those who stayed alone. Also, those respondents working in the laboratory and other workplaces were at a lower risk of anxiety than those working in a hospital.

Table S3 describes the relationship between risk factors for anxiety and adjustment for age, sex, religion, marital status, occupation, work experience, staying ‘alone or with family, friends or colleagues’, and working in the ICU and current workplace. The logistic regression analysis shows that female respondents were at a lower risk of depression than male respondents. Furthermore, respondents who had working experience for more than ten years were at a lower risk of depression than those working for less than two years.

## LITERATURE ON MENTAL HEALTH IMPACT AND SUPPORTIVE MEASURES DURING COVID-19 PANDEMIC

To the best of our knowledge, this is the first study investigating the immediate impact of COVID-19 pandemic on the mental health of healthcare workers from the six WHO regions. The data presented in this study provide evidence of a high prevalence of anxiety (60%) and depression symptoms (53%) among the healthcare workers across the regions. Similar findings for the symptoms of depression were reported among the healthcare workers in China as 50.4% [1]. Meanwhile, the prevalence of the symptoms of anxiety is higher in our study compared to the study conducted in China (44.6%) [1]. Our findings highlighted that healthcare workers from different regions have a substantial burden on mental health and warranted effective mental health support interventions. Diverse nature and workload of healthcare workers might have an influence on their psychological burden. However, the occupation was not associated with the anxiety and depression after adjusting all the demographic factors in the logistic regression model. This contrasts with the findings in China, where nurses were reported of having more severe symptoms compared to other professionals [1]. Working in the ICU and different workplaces were associated with the anxiety, suggesting that workplace might influence on the mental health impact among healthcare workers.

Healthcare workers who are staying alone during the COVID-19 pandemic, of Christian religion, and working in ICU are associated with more severe symptoms of anxiety. Our study further indicated that females, of Buddhist religion, and longer working experience (>10 years) were inversely associated with more severe symptoms of depression. This finding is in contrast to other works conducted in China [1] and Italy (Ref S6 in the **Online Supplementary Document**), where the female was more likely to report severe anxiety and depressive symptoms. In our study, males represented a higher proportion of doctors, working in hospitals, and having contact with COVID-19 confirmed or suspected cases compared to female healthcare workers, that might contribute to having a psychological burden. The junior healthcare workers with less than 2 years of working experience had higher depressive symptoms compared to those who were in the professional field for a longer duration (>10 years). This finding could be correlated with experience from Turkey, where the junior medical doctors were the main workforce during the COVID-19 pandemic (Ref S7 in the **Online Supplementary Document**). This situation might impose junior healthcare workers to be vulnerable, stressful, and had an impact on their mental health.

In terms of spirituality, human beings increased the tendency of returning to religion in the midst of a pandemic (Ref S8 in the **Online Supplementary Document**]. Dramatic increment on Google search for “prayer” [Ref S8 in the **Online Supplementary Document**), increment in the number of people praying to end the pandemic, including those who seldom or never prayed before, those who had no religious affiliation in American (Ref S9 in the **Online Supplementary Document**), and the practice of online worship were reported during the COVID-19 pandemic (Ref S10-S11 in the **Online Supplementary Document**). Future research should be conducted to explore the role of religion in coping with the psychological burden during the health crisis or pandemic.

The importance of mental health support for the healthcare workers during the COVID-19 pandemic has been emphasised by the WHO (Ref S12 in the **Online Supplementary Document**). Although the importance of mental health support for healthcare workers has been emphasised in different regions (Ref S13- S15 in the **Online Supplementary Document**), only one out of four healthcare workers in this study reported the availability of mental health support team at the workplace. Undoubtedly, an availability of a mental health support team is significantly associated with a lower prevalence of moderate to severe anxiety among the respondents. This finding can be related to the study conducted among healthcare workers in China, where the mental health support services curbed the acute psychological disturbances during COVID-19 (Ref S16 in the **Online Supplementary Document**).

The healthcare systems could address the urgent need for mental healthcare for healthcare workers through different innovative ways. Some interventions have been proposed to support the mental health of frontline healthcare workers such as quality-assured tele-counselling (Ref S17 in the **Online Supplementary Document**), or hotline mental health support from the external organizations. Meanwhile, interventions have been implemented, including digital mental health support packages, established for the frontline healthcare workers in the UK (Ref S18 in the **Online Supplementary Document**) and peer support project led by the mental health professionals by using the social media online chat groups (Ref S19 in the **Online Supplementary Document**). In our study, the majority of the respondents who had symptoms of anxiety and depression were of a mild to moderate severity category. Meanwhile, severe symptoms were reported in approximately 7% for anxiety, 3% for depression. Although the percentage is less, it is crucial to screen and provide support to severe cases to prevent adverse consequences. Further studies should investigate coping strategies among healthcare workers during the pandemic and the effectiveness of different mental health support strategies, that could provide valuable information for the organizations to set up an effective mental health support system.

In this study, the respondents were recruited with non-random convenience sampling method, and there might be a limitation in generalization of the findings. The questionnaire was distributed in English (except for Albania) and therefore, there is a potential bias of missing the respondents with low English-fluency. Other limitation pertains to the study instruments, GAD 7 and PHQ 9, that are self-administered instruments and therefore, further assessment by the clinician is beyond the scope of our study.

## CONCLUSION

During the COVID-19 pandemic, healthcare systems should address the psychological burden among healthcare workers. Mental health support for the healthcare workers should be available and accessible. Meanwhile, attention is needed for those who are staying alone, single, working in ICU, and those who have a shorter duration of working experience in their professions.

## Additional material

Online Supplementary Document
