# Supplementary material for: Immediate impact of COVID-19 on mental health and its associated factors among healthcare workers: A global perspective across 31 countries
Source: J Glob Health. 2020 Aug 23;10(2):020381. doi: 10.7189/jogh.10.020381 (PMC7649521; doi:10.7189/jogh.10.020381)
Supplement: Online Supplementary Document [file jogh-10-020381-s001.pdf]

Online Supplementary Document

Table S1 Socio-demographic characteristics and occupation of respondents (n=2097)

	**Doctor** (n=770) n(%)	**Nurse** (n=479) n(%)	**Others*** (n=848) n(%)	**Total** (n=2097) n(%)
**Sex**				
Male	369(47.9)	120(25.1)	200(23.6)	689 (32.9)
Female	401(52.1)	359(74.9)	648(76.4)	1408 (67.1)
**Age**				
<30 years	163(21.2)	224(46.8)	591(69.7)	978(46.6)
31-45 years	502(65.2)	178(37.2)	203(23.9)	883 (42.1)
46 years and above	105(13.6)	77(16.1)	54(6.4)	236 (11.3)
**Religion**				
Islam	291(37.1)	279(58.2)	703(82.9)	1273 (60.7)
Christian	419(54.4)	166(34.7)	109(12.9)	694 (33.1)
Buddhism	44(5.7)	6(1.3)	17(2.0)	67 (3.2)
Other†	16(2.1)	28(5.8)	19(2.2)	63 (3.0)
**Marital status**				
Married	527(68.4)	295(61.6)	479(56.5)	1301 (62.0)
Single	243(31.6)	184(38.4)	369(43.5)	796 (38.0)
**Work experience**				
<2 years	83(10.8)	106(22.1)	237(27.9)	426(20.32)
2-5 years	71(9.2)	113(23.6)	322(38.0)	506 (24.13)
6-10years	124(16.1)	85(17.7)	157(18.5)	366 (17.45)
>10years	492(63.9)	175(36.5)	132(15.6)	799 (38.10)
**Staying with (at the time of COVID-19 pandemic)**				
Alone	68(8.8)	23(4.8)	47(5.5)	138 (6.6)
Family	652(84.7)	408(85.2)	768(90.6)	1828 (87.2)
Friends/ colleagues	50(6.5)	48(10.0)	33(3.9)	131 (6.2)
**Working in the Intensive Care Unit (ICU)**				
No	588(76.4)	382(79.7)	802(94.6)	1772 (84.5)
Yes	182(23.6)	97(20.3)	46(5.4)	325 (15.5)
**Current workplace**				
Clinics	127(16.5)	118(24.6)	153(18.0)	398 (19.0)
Hospital	571(74.2)	295(61.6)	107(12.6)	973 (46.4)
Laboratory	23(3.0)	0(0.0)	35(4.1)	58 (2.8)
Pharmacy	1(0.1)	2(0.4)	74(8.7)	77 (3.7)
Others‡	48(6.2)	64(13.4)	479(56.5)	591 (28.1)

* Others included pharmacists, laboratory technicians, public health specialists, medical educators, and healthcare administrators

† Others included Hinduism, Shinto, and Atheists

‡ Others included healthcare institutions, Ministry of Health, Non-Governmental Organizations, rehabilitation centres

Table S2 Prevalence of anxiety and depression among the healthcare workers (n= 2097)

	**Occupation**					**Sex**				**Providing healthcare to COVID-19 confirmed cases or suspected cases**				**Availability of mental health support team at the workplace**			
**Anxiety**	Doctor n(%)	Nurse n(%)	Others * n(%)	Total n(%)	*P* †	Male n(%)	Female n(%)	Total n(%)	*P*†	No n(%)	Yes n(%)	Total n(%)	*P*†	No n(%)	Yes n(%)	Total n(%)	*P*†
Normal	245 (31.8)	211 (44.1)	375 (44.2)	831 (39.6)		217 (31.5)	614 (43.6)	831 (39.6)		603 (45.3)	228 (29.8)	831 (39.6)		623 (40.2)	208 (38.0)	831 (39.6)	
Mild	316 (41.0)	132 (27.6)	298 (35.1)	746 (35.6)		283 (41.1)	463 (32.9)	746 (35.6)		439 (33.0)	307 (40.1)	746 (35.6)		521 (33.6)	225 (41.1)	746 (35.6)	
Moderate	156 (20.3)	107 (22.3)	115 (13.6)	378 (18.0)		148 (21.5)	230 (16.3)	378 (18.0)		205 (15.4)	173 (22.6)	378 (18.0)		299 (19.3)	79 (14.4)	378 (18.0)	
Severe	53 (6.9)	29 (6.1)	60 (7.1)	142 (6.8)	<0.001	41 (6.0)	101 (7.2)	142 (6.8)	<0.001	85 (6.4)	57 (7.5)	142 (6.8)	<0.001	107 (6.9)	35 (6.4)	142 (6.8)	0.006
**Depression**																	
Normal	354 (46.0)	256 (53.4)	384 (45.3)	994 (47.4)		294 (42.7)	700 (49.7)	994 (47.4)		674 (50.6)	320 (41.8)	994 (47.4)		745 (48.1)	249 (45.5)	994 (47.4)	
Mild	263 (34.2)	92 (19.2)	244 (28.8)	599 (28.6)		208 (30.2)	391 (27.8)	599 (28.6)		343 (25.8)	256 (33.5)	599 (28.6)		441 (28.5)	158 (28.9)	599 (28.6)	
Moderate	85 (11.0)	89 (18.6)	133 (15.7)	307 (14.6)		127 (18.4)	180 (12.8)	307 (14.6)		192 (14.4)	115 (15.0)	307 (14.6)		216 (13.9)	91 (16.6)	307 (14.6)	
Moderately Severe	47 (6.1)	32 (6.7)	62 (7.3)	141 (6.7)		44 (6.4)	97 (6.9)	141 (6.7)		86 (6.5)	55 (7.2)	141 (6.7)		103 (6.6)	38 (6.9)	141 (6.7)	
Severe	21 (2.7)	10 (2.1)	25 (2.9)	56 (2.7)	<0.001	16 (2.3)	40 (2.8)	56 (2.7)	0.002	37 (2.8)	19 (2.5)	56 (2.7)	0.001	45 (2.9)	11 (2.0)	56 (2.7)	0.426
**Total**	770	479	848	2097		689	1408	2097		1332	765	2097		1550	547	2097	

* Others included pharmacists, laboratory technicians, public health specialists, medical educators, and healthcare administrators

† χ^2, $P < 0.05$ is considered as significant at 5 percent levels.

Table S3 Predictors of Anxiety and Depression through logistics regression analysis

		Anxiety				Depression			
		Adjusted OR	95% C.I.		P^*	Adjusted OR	95% C.I.		P^*
			Lower	Upper			Lower	Upper	
Sex	Male	Reference				Reference			
	Female	0.974	0.774	1.227	0.824	0.708	0.561	0.892	0.003
Age	<30 years	Reference				Reference			
	31-45 years	0.858	0.583	1.262	0.437	1.057	0.733	1.523	0.767
	46 years and above	1.244	0.760	2.037	0.385	1.587	0.965	2.609	0.069
Religion	Buddhism	Reference				Reference			
	Islam	1.583	0.778	3.222	0.206	2.887	1.280	6.513	0.011
	Christian	2.953	1.451	6.008	0.003	3.110	1.369	7.064	0.007
	Other	1.258	0.490	3.229	0.633	1.968	0.706	5.487	0.196
Marital status	Married	Reference				Reference			
	Single	1.009	0.787	1.295	0.941	1.325	1.035	1.697	0.026
Occupation	Doctor	Reference				Reference			
	Nurse	1.191	0.897	1.582	0.228	1.309	0.977	1.754	0.071
	Other	1.157	0.832	1.608	0.387	1.186	0.859	1.636	0.300
Work experience	<2 years	Reference				Reference			
	2-5 years	0.910	0.660	1.252	0.561	0.789	0.584	1.067	0.124
	6-10years	0.920	0.613	1.382	0.689	0.947	0.647	1.384	0.777
	>10 years	0.841	0.523	1.352	0.475	0.462	0.292	0.733	0.001
Staying with	Alone	Reference				Reference			
	Family	0.546	0.372	0.800	0.002	0.833	0.554	1.254	0.381
	Friends/ colleagues	0.481	0.277	0.836	0.009	1.073	0.618	1.862	0.802
Working in the (ICU)	No	Reference				Reference			

	Yes	1.685	1.274	2.228	0.000	1.337	0.989	1.809	0.059
Current workplace	Hospital	Reference				Reference			
	Clinics	0.905	0.669	1.225	0.518	1.319	0.973	1.788	0.075
	Laboratory	0.376	0.163	0.867	0.022	0.637	0.296	1.368	0.247
	Pharmacy	0.869	0.475	1.590	0.649	1.535	0.878	2.685	0.133
	Others	0.654	0.465	0.920	0.015	1.035	0.742	1.443	0.840

[*] Logistic regression analysis, $P < 0.05$ is considered as significant at 5 percent levels.

References

Ref S1. Adewuya AO, Ola BA, Afolabi OO. Validity of the patient health questionnaire (PHQ-9) as a screening tool for depression amongst Nigerian university students. J Affect Disord. 2006;96(1-2):89-93.

Ref S2. WHO. Process of translation and adaptation of instruments. 2015 [Available from: http://www.who.int/substance_abuse/research_tools/translation/en/]

Ref S3. Ahmad A. Health-Seeking Behavior and Its Determinants among Mine Workers in the Karauli District of Rajasthan in India. Dubai Med J 2019;2:7–16.

Ref S4. WHO. List of Member States by WHO Region and Mortality Stratum: World Health Organization; 2020 [Available from:https://www.who.int/choice/demography/mortality_strata/en/.]

Ref S5. The World Bank. World Bank Country and Lending Groups: World Bank Group; 2020 [Available from: https://datahelpdesk.worldbank.org/knowledgebase/articles/906519.]

Ref S6. Rossi R, Socci V, Pacitti F, Di Lorenzo G, Di Marco A, Siracusano A, et al. Mental Health Outcomes Among Frontline and Second-Line Health Care Workers During the Coronavirus Disease 2019 (COVID-19) Pandemic in Italy. JAMA Netw Open. 2020;3(5):e2010185.

Ref S7. Murt A. Junior doctors as the main health workforce of pandemic control hospitals in Turkey. Postgrad Med J. 2020.

Ref S8. Bentzen J. In Crisis, We Pray: Religiosity and the COVID-19 Pandemic. CEPR Discussion Paper No DP14824. 2020.

Ref S9. PewResearchCenter. Most Americans say coronavirus outbreak has impacted their lives 2020 [Available from: https://www.pewsocialtrends.org/2020/03/30/most-americans-say-coronavirus-outbreak-has-impacted-their-lives/.]

Ref S10. Newport F. Religion and the COVID-19 Virus in the U.S. Gallup. 2020.

Ref S11. Dein S, Loewenthal K, Lewis CA, Pargament KI. COVID-19, mental health and religion: an agenda for future research. Mental Health, Religion & Culture. 2020;23(1):1-9.

Ref S12. WHO. WHO calls for healthy, safe and decent working conditions for all health workers, amidst COVID-19 pandemic: World Health Organization; 2020 [Available from: https://www.who.int/news-room/detail/28-04-2020-who-calls-for-healthy-safe-and-decent-working-conditions-for-all-health-workers-amidst-covid-19-pandemic.]

Ref S13. Chersich MF, Gray G, Fairlie L, Eichbaum Q, Mayhew S, Allwood B, et al. COVID-19 in Africa: care and protection for frontline healthcare workers. Global Health. 2020;16(1):46.

Ref S14. Xiang YT, Jin Y, Wang Y, Zhang Q, Zhang L, Cheung T. Tribute to health workers in China: A group of respectable population during the outbreak of the COVID-19. Int J Biol Sci. 2020;16(10):1739-40.

Ref S15. Walton M, Murray E, Christian MD. Mental health care for medical staff and affiliated healthcare workers during the COVID-19 pandemic. Eur Heart J Acute Cardiovasc Care. 2020;9(3):241-7.

Ref S16. Kang L, Ma S, Chen M, Yang J, Wang Y, Li R, et al. Impact on mental health and perceptions of psychological care among medical and nursing staff in Wuhan during the 2019 novel coronavirus disease outbreak: A cross-sectional study. Brain Behav Immun. 2020.

Ref S17. UnitedNations. Policy Brief: COVID-19 and the Need for Action on Mental Health. United Nations; 2020.

Ref S18. Blake H, Bermingham F, Johnson G, Tabner A. Mitigating the Psychological Impact of COVID-19 on Healthcare Workers: A Digital Learning Package. Int J Environ Res Public Health. 2020;17(9).

Ref S19. Cheng P, Xia G, Pang P, Wu B, Jiang W, Li YT, et al. COVID-19 Epidemic Peer Support and Crisis Intervention Via Social Media. Community Ment Health J. 2020;56(5):786-
